# Human Milk Oligosaccharides Are Present in Amniotic Fluid and Show Specific Patterns Dependent on Gestational Age

**DOI:** 10.3390/nu14102065

**Published:** 2022-05-14

**Authors:** Evelyn Jantscher-Krenn, Lara von Schirnding, Martin Trötzmüller, Harald Köfeler, Una Kurtovic, Herbert Fluhr, Andreas Müller, Soyhan Bagci

**Affiliations:** 1 Department of Obstetrics and Gynecology, Medical University of Graz, 8036 Graz, Austria; una.kurtovic@medunigraz.at (U.K.); herbert.fluhr@medunigraz.at (H.F.); 2 BioTechMed, 8010 Graz, Austria; harald.koefeler@medunigraz.at; 3 Neonatology and Pediatric Intensive Care, Children’s Hospital, University of Bonn, D-53113 Bonn, Germany; laravonschirnding@gmail.com (L.v.S.); a.mueller@ukbonn.de (A.M.); 4 Core Facility Mass Spectrometry, Center for Medical Research, Medical University of Graz, 8036 Graz, Austria; martin.troetzmueller@uniklinikum.kages.at

**Keywords:** pregnancy, human milk oligosaccharides (HMOs), amniotic fluid, preterm nutrition, 2′-fucosyllactose, lactose, amniocentesis, fetal surgery

## Abstract

(1) Background: Human milk oligosaccharides (HMOs) are already found in maternal circulation in early pregnancy, changing with gestational age. HMOs are also present in cord blood and amniotic fluid (AF). We aimed to assess HMO profiles in AF over the course of gestation. (2) Methods: AF was collected during diagnostic amniocentesis, fetal surgery, or C-section from 77 women with a gestational age of ranging from 14.3 to 40.9 weeks. Samples were analysed using high performance liquid chromatography with fluorescence detection. (3) Results: We found lactose and up to 16 HMO structures in all AF samples investigated, starting at 14 weeks of gestation. Overall, 3′-sialyllactose (3′SL) and 2′-fucosyllactose (2′FL) were the most abundant HMOs. Individual and total HMO concentrations were significantly positively correlated with gestational age. HMO composition also changed between early, mid- and late pregnancy, with relative concentrations of 3′SL significantly decreasing (44%, 25%, 24%) and 2′FL increasing (7%, 13%, 21%), respectively. (4) Conclusion: Our study shows that HMOs are already present in AF early in pregnancy. This demonstrates extensive contact of the fetus with a broad variety of HMOs, suggesting roles for HMOs in fetal tissue development during the time course of pregnancy.

## 1. Introduction

Human milk oligosaccharides (HMOs) are complex, non-digestible human specific glycans that constitute the third-largest group of macromolecules in human milk, after lactose and lipids [1]. The concentration of HMOs depends on the stage of lactation, being highest in colostrum (22–24 g/L) and then dropping as milk matures (12–13 g/L) [2,3,4,5]. Over 150 different HMOs have been identified, composed of the five monosaccharides glucose, galactose, N-acetylglucosamine, fucose and sialic acid [6]. A common building block of all HMOs is lactose, a disaccharide produced by the lactating mammary gland.

In human milk, HMO composition and concentration vary inter-individually as well as intra-individually over time. Polymorphisms in two genes, the Secretor and Lewis genes, lead to varying activity in the encoded fucosyltransferases, FUT2 and FUT3, respectively, and thus to distinct fucosylation patterns, explaining part of the inter-individual variation [7,8]. Lactating mothers with an inactive FUT2 enzyme, so-called non-secretors, lack 2′-fucosyllactose (2′FL) and other α1-2 fucosylated HMOs in their milk. In addition to these genetic factors, environmental factors such as maternal nutritional and metabolic status are thought to influence concentration and composition of HMOs [4,9,10,11].

HMOs have many health-promoting effects on the infant. Their prebiotic [12] and antiadhesive/antimicrobial effects might mitigate the risk of infections of the gastrointestinal, respiratory and urinary tracts [13,14,15,16] in term and preterm infants. Specific HMOs are thought to prevent the development of necrotizing enterocolitis (NEC), one of the most common and serious comorbidities in very-low-birth-weight (VLBW) preterm infants [17,18,19]. Animal studies have suggested that sialylated HMOs, potentially by providing sialic acid as building block for brain gangliosides, promote neuronal and brain development and might have long-lasting effects on the development of cognitive functions [20,21]. Moreover, several studies have suggested HMO effects on intestinal barrier function, mucosal immune maturation and neonatal intestinal development [22,23,24].

Increasing evidence shows that the occurrence of HMOs is not limited to human milk and lactation. Using a high performance liquid chromatography (HPLC)-based method, we have previously shown that HMOs are already produced during pregnancy and are circulating in the maternal blood as early as the first trimester [25,26]. Serum HMOs are excreted via maternal urine during pregnancy [27,28]. While their potential roles in pregnancy remain elusive, varying HMO profiles might convey varying protective effects on mother and fetus, analogous to the effects of HMOs in breast milk on the nursing infant. Interestingly, higher serum concentrations of the HMO 3′SL during gestational weeks 23–34 were associated with risk of preterm delivery [27]. HMOs were also detected in the cord blood [29] as well as in the amniotic fluid (AF) of term-born infants [30]. However, it remains unclear when HMOs first appear in AF, and thus to what extent the fetus is exposed to HMOs prenatally, and whether and how concentration and composition of HMOs change during gestation.

AF is a dynamic and complex mixture of nutritive and bioactive factors, many of which are also found in human milk. Analogous to changes in human milk composition according to lactation stage and infant development, AF composition changes with pregnancy progression and fetal developmental status [31]. Bioactive factors in AF promote the development and maturation of fetal tissues exposed to AF in utero, including skin, lungs, kidneys and gastrointestinal (GI) tract [32]. At 16 weeks of gestation, the fetus starts swallowing AF. During late gestation, the fetus swallows up to 700 mL [33], about 80% of the total volume of AF, every 24 h. Preterm birth disrupts this constant exposure to AF factors. Thus, supplementation of AF in preterm infants has been suggested as a beneficial strategy to prevent potentially fatal disorders such as NEC in preterm infants at risk [34].

Based on these previous findings on prenatal HMOs in maternal serum [25,26], urine [27] and neonatal cord blood [29], we hypothesized that maternal HMOs passing to the fetus are excreted with fetal urine and are detectable in AF. With increasing circulating maternal HMOs and increasing fetal urine production over the course of gestation, we expected increasing concentrations of HMOs in AF. We here aimed to assess HMO composition and concentrations in AF and to investigate potential temporal changes with gestational age.

## 2. Materials and Methods

### 2.1. Human Subjects

Seventy-seven women admitted to the University Hospital Bonn between 2013 and 2018 were included in the study. The mean age ± SD (range) of the women was 33 ± 5 (19–47) years and mean gestational age at sampling was 34 ± 6.9 (14.3–40.9) weeks. In accordance with the Declaration of Helsinki, the study was approved by the Ethics Committee of the University of Bonn (077/13). We obtained written informed consent from all the women. Pregnant women with chorioamnionitis, premature rupture of membrane or a fetus with bilateral renal agenesis were excluded.

Amniotic fluid was obtained either during diagnostic amniocentesis (*n* = 3), fetal surgery (*n* = 9), or caesarean delivery (*n* = 65). Amniotic fluid samples were obtained by needle aspiration that was visually free of blood or meconium contamination. The amniotic fluid samples were centrifuged at 3000× *g*, aliquoted and stored at −80 °C until analysis. Gestational age was evaluated using the last menstrual period and ultrasonographic fetal biometry. Preterm infants are defined as newborns with a gestational age below 37 weeks of pregnancy. We collected 36 AF samples at gestational ages below 37 weeks either during ongoing pregnancies (via amniocentesis or fetal surgery) or from preterm deliveries, and 41 from full-term infants (C-sections).

### 2.2. Human Milk Oligosaccharide Standards

2′-Fucosyllactose (2′FL), 3-fucosyllactose (3FL), lacto-N-tetraose (LNT), lacto-N-neotetraose (LNnT), lacto-N-fucopentaose 1, 2, and 3 (LNFP 1, 2, and 3), lacto-N-difucohexaose 1 (LNDFH1), and lacto-N-hexaose (LNH) were purchased from Dextra Laboratories, Reading, United Kingdom. Lactodifucotetraose (LDFT), 3′-sialyllactose (3′SL), 6′-sialyllactose (6′SL), 3′sialyllactosamine (3′SLN), 6′-sialyllactosamine (6′SLN), sialyl-lacto-N-tetraose (LST) a, b, c, and disialyllacto-N-tetraose (DSLNT) (Glycoset II) were purchased from Prozyme, Hayward CA. Linear B6-Trisaccharide (Dextra Laboratories) was used as internal standard.

### 2.3. HMO Isolation and Analysis by HPLC

For initial HPLC runs, AF samples were pooled (*n* = 8) for three groups of gestational age ranges: early (14.0–21.9 weeks of gestation; when delivered would not be considered viable), mid- (22.0–33.9 weeks of gestation; when delivered, infants would be considered extremely to very preterm) and late pregnancy (34.0–41.0 weeks of gestation, corresponding to late preterm and term gestational ages). Samples were selected to be evenly distributed across the respective gestational age ranges. After confirming peaks by enzymatic digest and MS, HMOs were quantified in individual AF samples using HPLC with fluorescence detection. Oligosaccharides were isolated from amniotic fluid samples, with a protocol previously described for human serum [25]. In brief, AF samples with added internal standard (Linear B6-Trisaccharide) were worked up by removing lipids, proteins, and salts, using Chloroform/MeOH and solid phase extraction (SPE). Recovered, dried HMOs from AF samples were fluorescently labelled with 2-aminobenzamide (2AB) [19]. The 2AB-glycans were separated by HPLC with fluorescence detection on a TSKgel Amide-80 column (Tosoh Bioscience, Tokyo, Japan). Standard retention times were used to annotate HPLC peaks. The amount of each individual HMO was calculated based on normalization to internal standard. The relative abundance of each of the individual HMOs was determined by setting the sum of the 18 identified oligosaccharides as 100% total HMOs. HMO peak annotation by comparison with the retention times of commercially available HMO standards was confirmed by exoglycosidases and LC-MS.

### 2.4. Analysis of HMOs by Enzymatic Digest

To confirm the identity of individual peaks in AF, 2AB-labelled samples were treated either with α2-3 neuraminidase (New England Biolabs, #P0743L), α2-3,6,8 neuraminidase (New England Biolabs #P0720S) or α1-2-fucosidase (New England Biolabs, #P0724S) according to the manufacturer’s instructions. Enzyme-treated and untreated samples were run in parallel and analyzed by HPLC with fluorescence detection as described above.

### 2.5. Determination of Oligosaccharides by LC-MS

Pooled AF samples were prepared as described for the HPLC method (*n* = 8) for the three groups of gestational age ranges: early (14.0–21.9 weeks of gestation), mid- (22.0–33.9 weeks of gestation) and late pregnancy (34.0–41.0 weeks of gestation. An HPLC (Thermo Fisher Scientific, Waltham, MA, USA) with a TSKgel Amide-80 (Tosoh Bioscience, Tokyo, Japan) and a linear gradient of a 50 mmol/L-ammonium formate/acetonitrile solvent system was used. Oligosaccharides were determined by an Orbitrap in positive ESI mode. The spray voltage was set to 4000 V, capillary voltage to 35 V and vaporizer temperature was 250 °C.

### 2.6. Statistics

The statistical analyses were performed using GraphPad Prism (version 9.1.2.; GraphPad Software, La Jolla, CA, USA). Data are presented as means ± standard deviation (range). HMO concentrations are presented as medians (interquartile range, IQR) in nmol/mL. The variables were tested for normality using the Kolmogorov-Smirnov test. Spearman correlation analyses were used to analyze associations among HMO concentrations, gestational age, body mass index (BMI) and maternal age. For all analyses, *p*-values of less than 0.05 were considered statistically significant.

Concentrations of individual and total oligosaccharides were normalized to the internal standard linear B6 Trisaccharide. Relative abundances were calculated as the percentage of an individual HMO of the summed unambiguously identified oligosaccharides (total HMOs). 3FL was excluded from quantifications and all calculations, due to a co-elution with an unidentified peak. Due to the low concentration of the peak containing 3FL, no further investigation of the unknown coeluting peak was pursued. 6′SL coeluted with an unidentified oligosaccharide of known mass (annotated as Hex4) and was analyzed as combined peak 6′SL/Hex4. Data for total HMOs did not include 3FL and 6′SL/Hex4. 

Changes in HMOs concentration between the 3 groups (early pregnancy, 14.0–21.9 weeks; mid-pregnancy, 22.0–33.9 weeks; and late pregnancy, 34.0–41.0 weeks of gestation) were tested using ANOVA (Kruskal–Wallis test with Dunn’s multiple comparison), and graphs were plotted in GraphPad Prism (version 9.1.2.; GraphPad Software, La Jolla, CA, USA).

## 3. Results

### 3.1. HMO Profiles in Amniotic Fluid in Early, Mid and Late Pregnancy

**Figure 1:**
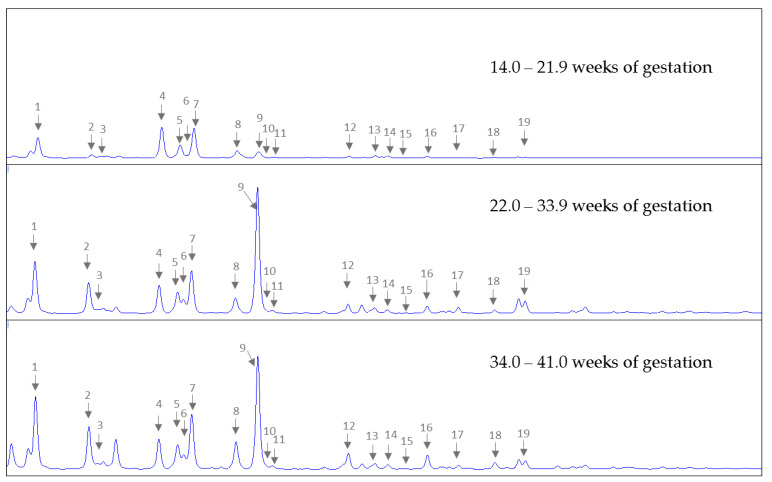
Representative chromatograms of pooled AF sampled at different periods during pregnancy. Eight samples each from between 14.0–21.9 weeks (**upper panel**), 22.0–33.9 weeks (**middle panel**) or 34.0–41.0 weeks of gestation (**lower panel**) were worked up and pooled for HPLC analysis. (1) lactose; (2) 2′-fucosyllactose (2′FL); (3) 3-fucosyllactose (3FL); (4) internal standard; (5) 3′-sialyllactosamine (3′SLN); (6) difucosyllactose (LDFT); (7) 3′-sialyllactose (3′SL); (8) 6′-sialyllactosamine (6′SLN); (9) 6′-sialyllactose (6′SL); (10) lacto-N-tetraose (LNT); (11) lacto-N-neotetraose (LNnT); (12) lacto-N-fucopentaose (LNFP1); (13) lacto-N-fucopentaose (LNFP2,3); (14) sialyl-lacto-N-tetraose (LST)a; (15) LSTb; (16) LSTc, (17) lacto-N-difucohexaose (LNDFH); (18) lacto-N-hexaose (LNH); (19) disialyllacto-N-tetraose (DSLNT).

To obtain an overview of HMO profiles in AF, we allocated the AF samples into three sampling periods according to stage of pregnancy, ie., early pregnancy (14.0–21.9 weeks; range 14.3–21.7 weeks, median 19 weeks; *n* = 8), mid pregnancy (22.0–33.9 weeks; range 22.6–32.4 weeks, median 29.0 weeks; *n* = 15) and late pregnancy (34.0–41.0 weeks, range 34.1–40.9, median 38.0 weeks; *n* = 54), and pooled eight individual samples for each pregnancy stage. HPLC analysis of these pooled AF samples revealed the presence of up to 16 HMOs (2′FL, 3FL, 3′SL, 6′SL, LNT, LNnT, 3′S-3FL, LNFP1, 2, 3, LSTa, b, c, LNDFH, LNH, DSLNT) which had all been previously reported in maternal serum in pregnancy. We also annotated the lactosamines 3′SLN and 6′SLN, two oligosaccharides present in maternal serum during pregnancy not usually present in human milk. Figure 1 shows representative HPLC chromatograms of HMOs isolated from pooled AF samples from early, mid- and late pregnancy. We performed an enzymatic digest using these pooled samples to confirm peaks identified by standard retention times.

Treatment with either α1-2 fucosidase or α2-3 neuraminidase resulted in the removal or major reduction of the expected peaks (2′FL, LDFT, LNFP1 and LNDFH for α1-2 fucosidase, and 3′SL and 3′SLN for α2-3 neuraminidase, respectively) (data not shown). However, pan-sialidase (α2-3,6,8 neuraminidase), which catalyzes the hydrolysis of all sialylated linkages, did not reduce the peak preliminary annotated as 6′SL, which was highly abundant in pools from mid- and late pregnancy. We used LC-MS on the same 2-AB-labelled pooled HPLC samples to confirm the respective masses for all 18 peaks assigned by retention times of HMO standards. All masses of HMOs were confirmed at the respective retention times (Supplementary Figure S1). However, 6′SL had a relatively low intensity, unlikely to account for the dominant peak in the HPLC chromatograms. Coinciding with the retention time of 6′SL, we found a higher abundant signal with mass of 787.29 corresponding to the 2AB H+ adduct of a tetrasaccharide containing 4 hexoses (Supplementary Figure S2).

### 3.2. HMO Concentrations and Composition Change with Gestational Age

**Figure 2:**
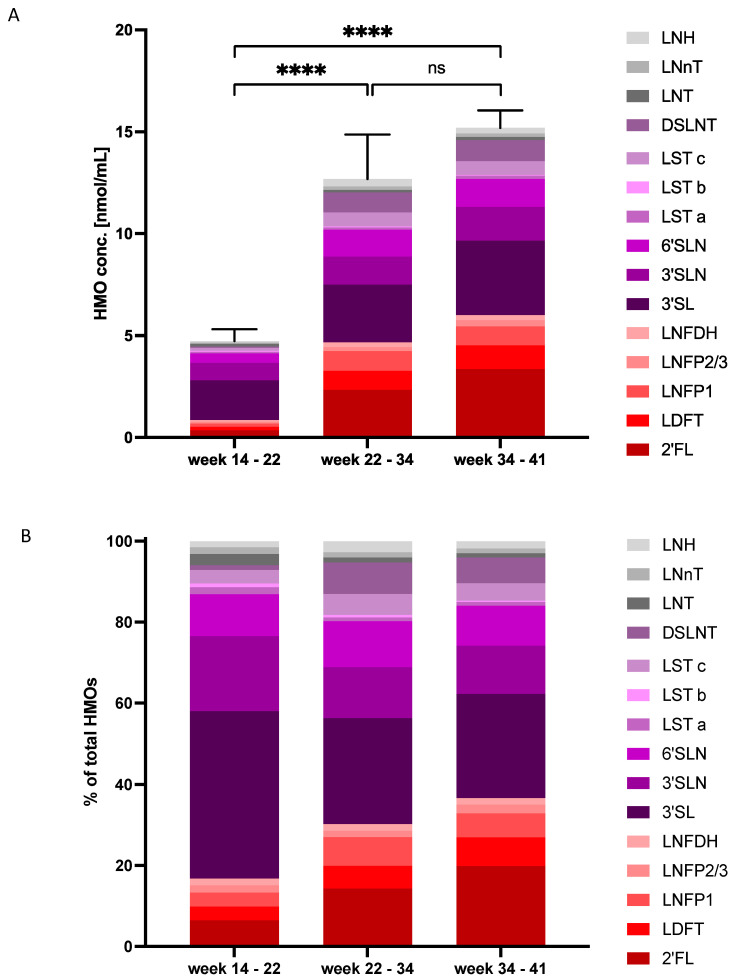
HMO concentrations and composition change with gestational age. AF samples from early (14.0–21.9 weeks, *n* = 8), mid- (22.0–33.9 weeks, *n* = 15) and late (34.0–41.0 weeks, *n* = 54) pregnancy were individually measured by HPLC. (**A**) Means ± SEM of total HMO concentrations (in nmol/mL) in the respective sampling periods were significantly different (Kruskal–-Wallis test with Dunn’s multiple comparisons test). Stacked bars show the means of unambiguously identified HMOs (without 3FL and 6SL/Hex4) for the three sampling periods. ns, not significant; **** *p* < 0.0001. (**B**) HMO composition changes with gestational age. Stacked bars show mean relative concentrations of unambiguously identified HMOs for the different sampling periods. Fucosylated HMOs (2′FL, LDFT, LNFP1, LNFP2/3, LNDFH) are colored in shades of red, sialylated HMOs (3′SL, 3SLN, 6′SL, 6′SL, LSTa, LSTb, LSTc, DSLNT) in shades of purple, and unmodified HMOs (LNT, LNnT, LNH) in shades of gray.

We next analyzed individual AF samples by HPLC to investigate HMO concentrations and composition, and temporal changes thereof. Table 1 shows maternal characteristics and sampling information for the total group of 77 women, grouped into respective gestational age periods at sampling: early, mid- and late pregnancy. The groups did not significantly differ in maternal age or BMI. In all AF samples investigated, we found lactose and HMOs, starting at as early as 14 weeks of gestation. Retention times of low abundant 3FL and 6′SL coincided with two others, not yet identified peaks, interfering with the quantification of these peaks by HPLC. LNFP2 and LNFP3 did not fully separate and were quantified together. Table 2 shows the median concentrations (with IQR) of lactose and individual HMOs unambiguously identified for the three gestational periods. Because 6′SL and Hex4 could not be distinguished they were quantified together. Most individual and total concentrations of HMOs significantly increased from early to late pregnancy. The greatest absolute increase was found for the combined peak 6′SL/Hex4 (0.45 nmol/mL, 7 nmol/mL and 6.1 nmol/mL) and for 2′FL (0.27 nmol/mL, 1.2 nmol/mL, 3.1 nmol/mL) from early to mid- and late pregnancy, respectively. Total HMO concentration significantly increased from early to mid- and late pregnancy (Figure 2A).

The composition of HMOs also significantly changed during the course of pregnancy; relative concentrations of 3′SL significantly decreased from 43.5% to 24.5% and 23.6%, and 2′FL increased from 6.7% to 12.5% and 21.0%, respectively (Figure 2B). After the 22nd week, higher concentrations of more complex HMOs such as LNFP1 and DSLNT were detectable. Overall, the secretor-dependent HMOs, 2′FL and LDFT showed the largest variations within the gestational age groups. In approximately 20% of all samples in mid- (22–34 weeks) and late (34–41 weeks) pregnancy, we found very low concentrations of these HMOs, indicating these samples were stemming from secretor-negative women. Interestingly, the peak corresponding to 6′SL and an unidentified tetrasaccharide with the mass of 4 hexoses showed the highest median concentration in AF of mid- and late pregnancy samples. In early pregnancy, 3′SL was the HMO with the highest concentration.

### 3.3. HMOs Are Highly Correlated with Each Other

**Figure 3:**
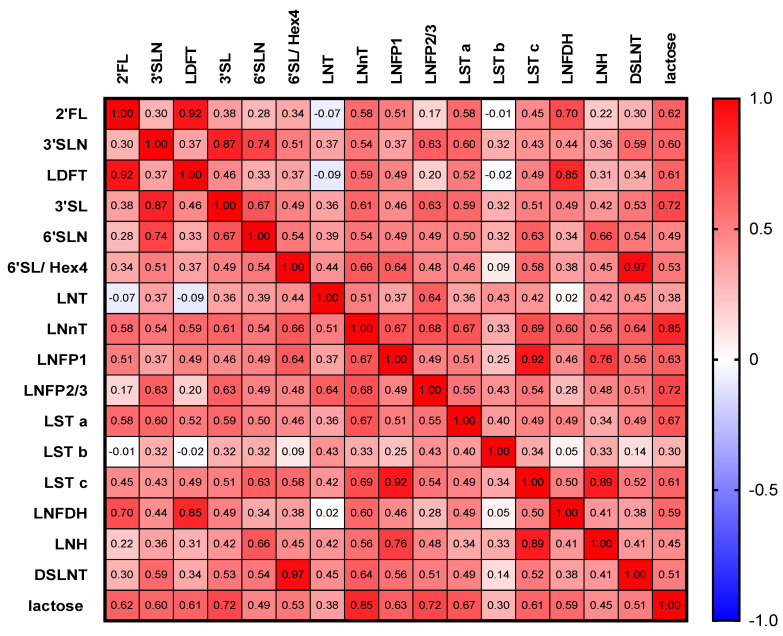
Intercorrelation of HMOs with each other across all sampling periods. The correlation matrix shows Spearman correlations (positive correlations in red, negative correlations in blue) of individual HMOs with each other. All correlations with Spearman coefficient r > 0.17 were significant or highly significant.

Most HMOs were significantly positively correlated with each other, when considered across all sampling periods (early, mid and late pregnancy, *n* = 77) (Figure 3). We found the strongest positive correlation within fucosylated HMOs, 2′FL vs. LDFT (r = 0.92, *p* < 0.0001), 2′FL vs. LNDFH (r = 0.7, *p* < 0.0001), and LDFT vs. LNDFH (r = 0.85, *p* < 0.0001) and within the sialylated HMOs, e.g., 3′SL vs. 3′SLN (r = 0.87, *p* < 0.0001) and 6′SLN (r = 0.74, *p* < 0.0001). Lactose showed the highest correlations with LNnT (r = 0.85, *p* < 0.0001), LNFP2/3 (r = 0.72, *p* < 0.0001) and 3′SL (r = 0.72, *p* < 0.0001).

### 3.4. HMOs in Amniotic Fluid Are Associated with Maternal Factors

To investigate whether HMOs in AF are associated with maternal factors, we performed association studies with available data including maternal BMI, maternal age and gestational age at sampling. Lactose and most of the individual HMOs as well as the total sum of HMOs were significantly positively correlated with gestational age (Table 3). Secretor-status-dependent fucosylated HMOs 2′FL, LDFT or LNDFH showed weaker correlations (Spearman coefficients, r = 0.35, r = 0.37 and r = 0.43; *p* < 0.0001). Highest correlations were observed for 3′SLN and LNFP2/3 with gestational age (r = 0.56 and r = 0.57, respectively), similar to lactose (r = 0.56, *p* < 0.0001). Weaker correlations found for HMOs in AF and maternal age (3′SL and 6′SLN) or BMI (LNFP1) (Table 3) disappeared when correcting for gestational age (not shown).

## 4. Discussion

Our study showed that lactose and HMO species, previously also found in maternal pregnancy serum and urine, as well as in cord blood, are present in AF during pregnancy. Similar to previous results for serum and urine, HMOs appear in AF as early as the beginning of the second trimester. These findings demonstrate extensive contact of the fetus with a broad variety of HMOs in utero, pointing to HMOs as potential modulating factors for fetal development. Given that the fetus is submerged in and infused by HMOs, this finding could be especially valuable for developing therapeutic concepts in the future for prematurely born infants who will otherwise miss out on this extensive contact with HMOs.

### 4.1. Occurrence of Uterine HMOs and Lactose

We found 16 oligosaccharides previously also detected in maternal blood and urine during pregnancy [25,26,27]. We also found lactose, a prerequisite for HMO production, to be relatively prominent in amniotic fluid samples. Interestingly, the existence of lactose in human amniotic fluid has not yet been reported. Wise et al. [30] had previously reported four HMOs (2′FL, 3FL, LDFT, 6′SL) in a small study of eight amniotic fluid samples collected at term births. The authors also reported a peak with retention time of 6′SL showing a higher relative abundancy than in breast milk. Using mass spectrometry (MS), they confirmed a mass corresponding to 6′SL, but due to sensitivity issues tandem MS did not demonstrate 6′SL. This seems to fit well with our observation that 6′SL is present, but due to the co-elution of another oligosaccharide, not in the abundance that the HPLC peak might suggest.

### 4.2. HMO Concentrations in AF over Gestational Periods

We here report, for the first time, HMOs in amniotic fluid samples from different time periods ranging from as early as 14 weeks of gestation up to full term. Most HMOs in AF were positively correlated with gestational age, and we found changes in HMO composition across the three sampling periods. This fits very well with our previous studies of maternal serum, showing very similar changes in concentrations of individual HMOs, especially secretor-dependent HMOs such as 2′FL and LDFT, and an overall increase in the relative abundance of fucosylated HMOs over time [25]. In future studies, it would be interesting to correlate the individual HMOs in AF with paired maternal serum samples from the same sampling time point.

For temporal analyses, we grouped the AF samples into early, mid- and late pregnancy according to gestational weeks 14.0–21.9, 22.0–33.9 and 34.0–41.0. We chose this grouping as it corresponds to gestational ages at which the fetus, when delivered, would either be considered to have low chance of survival (before 22.0 weeks), or be born extremely to very preterm (22.0–33.9 weeks), or moderately preterm to term (34.0–41.0 weeks of gestation). In the second half of pregnancy, most HMOs in AF did not significantly change between mid- and late pregnancy, and the largest increases in HMO concentrations were found from the sampling period below 22 to the period beyond 22 weeks of gestation. This finding of already relatively high HMO concentrations in AF in gestational weeks 22–34 suggests that extremely to very preterm infants might miss out on several weeks of extensive intrauterine HMO exposure.

### 4.3. Origin of Uterine HMOs

**Figure 4:**
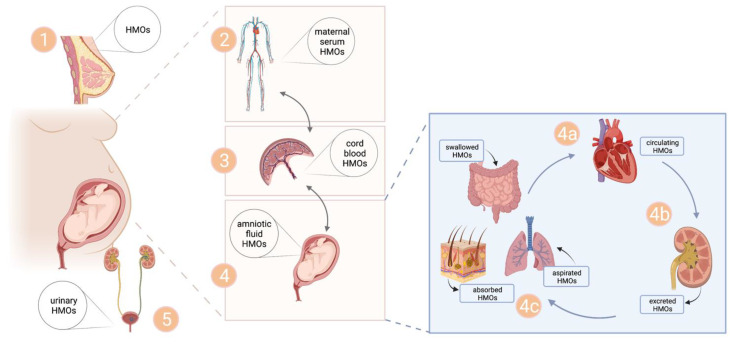
Scheme of the proposed pathway of HMOs in pregnancy. HMOs are produced in the mammary gland (1) during pregnancy, reach the maternal circulation (2) and might cross the placental barrier to reach the cord blood (3) and the amniotic fluid (4), before they are excreted via maternal urine (5). HMOs enter fetal circulation via the umbilical vein, and might either cycle back to the placenta via the umbilical artery or be excreted via fetal urine to reach amniotic fluid (AF) (4a–4c). AF HMOs are swallowed and come in extensive contact with gastrointestinal tract mucosal lining, where they are partly absorbed into fetal blood (4a) and are excreted via urine (4b). HMOs in AF might potentially be absorbed by fetal skin and might also come into contact with lungs via fetal aspiration of AF. Created with BioRender.com.

In a previous study, we found HMOs in cord blood and demonstrated in an ex-vivo placental perfusion study that 2′FL can be transferred from the maternal to the fetal circuit [29]. These findings suggested that maternal circulating HMOs cross the placental barrier and reach fetal blood. HMOs in fetal circulation might be excreted largely via fetal urine. Given that a significant part of AF consists of fetal urine, it seems likely that HMOs in the AF stem from renal excretion of the fetus, which usually starts at the 13th gestational week and might accumulate in the uterine compartment. In AF from gestational weeks 34–41, we found total HMO concentrations to be about 15 times higher than HMO concentrations previously found in cord blood of term pregnancies, which amounted to about 1 nmol/mL. This ratio seems to be in line with the ratio of HMOs in maternal urine to HMOs in maternal blood, which was about 50 [27]. It cannot here be confirmed whether the maternal to fetal route via the placenta is the sole contributor to the HMOs in AF, or whether HMOs also directly stem from maternal plasma transfer across the amnion to the amniotic cavity. Figure 4 shows a scheme of the proposed route of HMOs from maternal blood via the placenta into fetal circulation and the uterine compartment, and depicts fetal epithelial tissues potentially exposed to HMOs in AF.

### 4.4. Exposure of Fetal Tissues to HMOs—Implicatitons of HMOs for Fetal Development?

Based on our finding that a complex mix of HMOs is present in AF in early pregnancy, several fetal tissues are potentially exposed to HMOs during fetal development, with unknown implications. However, it is tempting to speculate that the relatively high concentrations of HMOs in AF in mid- and late pregnancy promote maturation of the intestinal epithelial tissue. Our data suggest that in late pregnancy, when the fetus swallows about 700 mL of AF each day, more than 10 mg of HMOs pass the fetal GI tract. In a previous study, we found among newborns with esophageal atresia significantly higher rates of infants small for gestational age and with lower birth weight z-scores, compared with newborns with anorectal malformations. These findings suggest that enteral uptake of AF plays a major role in fetal growth [35]. Similarly, in a study of neonates suffering from gastrointestinal atresia in different parts of the GI tract, Burjonrappa et al. [36] suggested that length and surface area of intestinal exposure to AF are associated with higher birth weight. Immaturity of the intestine distal to atresia can be explained by lack of exposure to AF and trophic factors therein [37]. Previous in vitro studies have shown that neutral and acidic HMOs inhibit proliferation and induce differentiation in intestinal cell lines [38,39], processes important for the maturation of intestinal tissues. In this context, future studies will investigate individual and total HMOs in AF in association with fetal growth for gestational age in fetuses with and without atresia. However, HMOs in AF might be relevant not only to fetal growth and intestinal maturation, as other fetal surfaces and epithelial tissues come into contact with HMOs via AF, including the skin, and potentially also the lungs, through fetal breathing movements [40]. Thus, it will be interesting to investigate whether AF and the HMOs therein might also contribute to maturation of these tissues. In this line of thoughts, one might also speculate about HMO effects on the fetal urinary tract, as AF HMOs likely result from fetal urine.

### 4.5. Potential Clinical Implications for AF-Based Predictive, Preventive or Therapeutic Strategies?

A large body of evidence shows that HMOs contribute to the beneficial effects of human milk feeding for reducing necrotizing enterocolitis (NEC). Disialyllacto-N-tetraose (DSLNT), one of the HMOs that we also identified in AF, was previously shown effective in an NEC animal model [19]. Lower DSLNT levels in mothers’ own milk can predict increased risk of developing NEC in preterm VLBW infants [17]. In this regard, it would be interesting to test HMO signatures in AF at the birth of a VLBW baby as potential predictor of NEC development. Similar to human subjects, AF has also been shown to reduce gut inflammatory processes and NEC severity in mice [34] and preterm pigs [41]. Porcine or human AF decreased intestinal inflammatory cytokines and increased body weight [42], inviting speculation that HMOs account for these effects of AF, similar to direct HMO effects observed in vitro [22,23,43]. Thus, our findings may be valuable for developing future preventive approaches for infants at risk of VLBW. Considering other vulnerable tissues of extremely to very preterm infants, such as the skin and potentially the lungs, further preventive and therapeutic HMO-based strategies might emerge in the future. Extremely and very preterm born infants face multiple health problems due to the stark differences in environmental factors between intra and extrauterine life, and due to the immaturity of tissues such as gut, lung and skin. HMOs in AF might be considered as potential adjuvants in postnatal care of these vulnerable newborns to improve outcomes [32].

### 4.6. Strengths and Limitations

The strengths of this study are the wide range of gestational ages of the amniotic fluid samples (14–41 weeks of gestation), the relatively large sample size and the novelty of our findings of up to 16 different oligosaccharides and lactose.

There are however some limitations to our study. Obtaining AF samples from early and mid-pregnancy by amniocentesis and during fetal surgery (e.g., for feto-fetal transfusion syndrome) and medically indicated preterm C-sections (e.g. IUGR, PE) suggests that these pregnancies were complicated by diverse pathologies requiring an invasive therapeutic treatment. Thus, strictly speaking, some of these pregnancies cannot be considered physiologic, however, for obvious ethical reasons this cannot be circumvented. Although we cannot completely rule out that especially in early pregnancy HMO profiles in AF develop slightly differently from uncomplicated pregnancies, HMO dynamics closely resemble those previously reported in serum. Another limitation of this pilot study is that maternal material such as serum and urine from the same time points was not available, making it impossible to compare maternal HMO profiles with the AF profiles. Although not the scope of the study, it would have been interesting to have more detailed maternal clinical and physiological data or information on pregnancy outcomes, to assess associations with HMOs in AF. Larger studies will be needed to further investigate the influence of early amniotic fluid HMOs on fetal growth and neonatal outcomes.

## 5. Conclusions

Our findings highlight human “milk” oligosaccharides as novel constituents of the physiological uterine environment across gestation, potentially critical for maturation of fetal tissues exposed to HMOs. HMOs might hold potential as adjuvants in postnatal care of extremely and very preterm infants, and AF in the transition from fetal to postnatal nutrition can be further investigated as an alternative source of HMOs. The notion that HMO composition may affect not only the health of breast-fed newborns, but might already have implications on the health of the developing fetus, will set the stage for future nutritional strategies for extremely preterm infants.

## Figures and Tables

**Table 1:** Study characteristics.

Maternal Characteristics	Total Group	Early Pregnancy (14.0–21.9 Weeks of Gestation)	Mid Pregnancy (22.0–33.9 Weeks of Gestation)	Late Pregnancy; (34.0–41.0 Weeks of Gestation)	ANOVA *p* Value
	*n* = 77	*n* = 8	*n* = 15	*n* = 54	
Maternal age (years) ± SD	33.3 ± 5.4	32.8 ± 4.1	31.7 ± 5.5	33.8 ± 5.5	0.823
BMI (kg/m^2^) ± SD	28.4 ± 5.8	27.0 ± 4.0	26.1 ± 4.5	29.3 ± 6.1	0.126
Gestational age (weeks) [min–max]	34.0 [14.3–40.9]	18.3 [14.3–21.7]	28.1 [22.6–32.6]	37.8 [34.1–40.9]	
sample collection					
Fetal surgery (*n*)	9 (12%)	5 (63%)	4 (27%)	0 (0%)	
Amniocentesis (*n*)	3 (4%)	3 (38%)	0 (0%)	0 (0%)	
C-section (*n*)	65 (84%)	0 (0%)	11 (73%)	54 (100%)	
Twin pregnancies (*n*)	17 (22%)	5 (63%)	6 (40%)	6 (11%)	
IUGR/placenta insufficiency (*n*)	10 (13%)	1 (13%)	8 (53%)	1 (2)%	
HDP (*n*)	6 (8%)	0 (0)	1 (7%)	5 (9%)	
GDM (*n*)	7 (9%)	0 (0%)	0 (0%)	7 (13%)	
Primary C-section * (*n*)	19 (25%)	0 (0%)	2 (13%)	17 (31%)	

Means ± SD; HPD, hypertensive disorders in pregnancy; GDM, gestational diabetes; * re-sections and elective C-sections.

**Table 2:** Lactose and HMO concentrations in amniotic fluid during pregnancy (nmol/mL).

	Early Pregnancy (Weeks 14.0–21.9)		Mid Pregnancy (Weeks 22.0–33.9)		Late Pregnancy (Weeks 34.0–41.0)		
	(*n* = 8)		(*n* = 15)		(*n* = 54)		ANOVA
	Median	IQR	Median	IQR	Median	IQR	*p* Value
Lactose	1.561	0.995–1.792	2.598	1.572–5.190	4.512	3.826–5.603	<0.0001
2′FL	0.272	0.103–0.569	0.970	0.165–3.500	2.769 ^a^	0.754–5.362	0.0011
3′SLN	0.873	0.600–1.067	1.282	0.795–1.822	1.607 ^a^	1.318–1.891	0.0011
LDFT	0.129	0.074–0.311	0.422	0.108–1.667	0.959 ^a^	0.411–1.699	<0.0001
3′SL	1.801	1.282–2.379	2.355	1.803–3.781	3.379 ^a^	2.896–4.078	0.0003
6′SLN	0.472	0.397–0.565	1.225 ^a^	0.553–1.615	1.296 ^a^	1.024–1.499	<0.0001
6′SL/Hex4	0.458	0.357–0.670	6.981 ^a^	4.355–12.160	6.096 ^a^	4.090–12.680	<0.0001
LNT	0.114	0.088–0.164	0.101	0.066–0.218	0.101	0.081–0.167	0.9367
LNnT	0.074	0.065–0.082	0.152 ^a^	0.067–0.238	0.161 ^a^	0.113–0.214	0.0003
LNFP1	0.154	0.115–0.233	0.808 ^a^	0.164–1.32	0.681 ^a^	0.433–1.122	0.0002
LNFP2/3	0.078	0.045–0.120	0.085	0.065–0.282	0.266 ^a,b^	0.175–0.368	<0.0001
LSTa	0.073	0.057–0.102	0.089	0.059–0.114	0.128 ^a^	0.084–0.185	0.026
LSTb	0.040	0.031–0.053	0.051	0.037–0.081	0.040	0.028–0.060	0.1337
LSTc	0.131	0.101–0.224	0.570 ^a^	0.168–0.871	0.510 ^a^	0.355–0.800	0.0002
LNDFH	0.075	0.049–0.095	0.134	0.067–0.343	0.232 ^a^	0.111–0.344	0.0022
LNH	0.0623	0.044–0.100	0.261 ^a^	0.061–0.372	0.189 ^a^	0.124–0.304	0.0011
DSLNT	0.0437	0.040–0.070	0.669 ^a^	0.315–1.608	0.693 ^a^	0.361–1.162	<0.0001
fucosylated	0.676	0.459–1.322	1.998 ^a^	1.120–6.446	5.314 ^a^	2.703–8.519	<0.0001
sialylated	3.521	2.534–4.409	8.269 ^a^	4.434–10.660	7.980 ^a^	6.500–9.204	<0.0001
unmodified	0.266	0.193–0.341	0.559 ^a^	0.220–0.933	0.497 ^a^	0.349–0.803	0.0043
total HMOs	4.323	3.435–6.324	9.762 ^a^	6.698–15.390	14.740 ^a^	10.880–17.580	<0.0001

Kruskal–Wallis ANOVA with Dunn’s multiple comparison testing to reveal whether time (early, mid-, late pregnancy) had a significant effect on individual, grouped fucosylated, sialylated and unmodified or total HMO concentrations. Lower case superscripts indicate significance (*p* < 0.05) with the denoted time interval (^a^, vs. early pregnancy; ^b^, vs. mid pregnancy). Total HMOs refers to sum of all unambiguously identified HMO peaks (without 3FL and 6SL/Hex4).

**Table 3:** Spearman correlations between gestational age (in weeks), maternal age (in years), and maternal BMI (kg/m^2^), and individual and grouped HMOs (in nmol/mL).

	Gestational Age		Maternal Age		Maternal BMI	
	Spearman r	*p* Value	Spearman r	*p* Value	Spearman r	*p* Value
2′FL	0.35	0.0009	0.06	0.3065	−0.12	0.1585
3′SLN	0.56	<0.0001	0.19	0.0519	0.10	0.1963
LDFT	0.37	0.0004	0.09	0.2168	−0.05	0.3246
3′SL	0.48	<0.0001	0.29	0.0060	0.08	0.2550
6′SLN	0.35	0.001	0.25	0.0131	0.06	0.3154
6′SL/Hex4	0.3	0.0037	−0.13	0.1254	−0.07	0.2661
LNT	0	0.497	0.00	0.4928	−0.08	0.2465
LNnT	0.36	0.0007	0.11	0.1650	−0.08	0.2545
LNFP1	0.24	0.017	−0.02	0.4429	−0.27	0.0081
LNFP2/3	0.54	<0.0001	0.14	0.1089	0.00	0.4937
LST a	0.45	<0.0001	0.23	0.0206	−0.10	0.1870
LST b	−0.05	0.3416	0.14	0.1137	−0.11	0.1724
LST c	0.27	0.0093	0.05	0.3327	−0.18	0.0602
LNFDH	0.43	<0.0001	0.14	0.1140	−0.01	0.4533
LNH	0.18	0.0626	0.10	0.1944	−0.06	0.2913
DSLNT	0.3	0.0037	−0.09	0.2108	−0.02	0.4482
total HMOs *	0.46	<0.0001	0.15	0.0979	−0.08	0.2318
fucosylated	0.4	0.0002	0.07	0.2784	−0.15	0.1020
sialylated	0.44	<0.0001	0.18	0.0593	0.02	0.4241
unmodified	0.21	0.0305	0.09	0.2208	−0.08	0.2515
lactose	0.56	<0.0001	0.16	0.0869	−0.11	0.1737

* without 3FL, 6′SL/Hex4.

## Data Availability

Not applicable.

## Supplementary Materials

The following supporting information can be downloaded at: www.mdpi.com/article/10.3390/nu14102065/s1, Figure S1: Confirmation of HMOs in AF by mass spectrometry. Figure S2: Detection of unidentified oligosaccharide at retention time of 6′SL.

## Abbreviations

Amniotic fluid (AF); Human milk oligosaccharide (HMO); 2′-Fucosyllactose (2′FL); lacto-difucotetraose (LDFT); 3′-sialyllactose (3′SL); 3′-sialyllactosamine (3′SLN); 6′-sialyllactosamine (6′SLN); lacto-N-tetraose (LNT); lacto-N-neo-tetraose (LNnT); lacto-N-fucopentaose (LNFP); lacto-N-difucohexaose 1 (LNDFH1), lacto-N-hexaose (LNH); sialyl-lacto-N-tetraose (LST); disialyl-lacto-N-tetraose (DSLNT); hypertensive disorders of pregnancy (HDP); gestational diabetes mellitus (GDM).
