# Supplementary material for: Human Milk Oligosaccharides Are Present in Amniotic Fluid and Show Specific Patterns Dependent on Gestational Age
Source: Nutrients. 2022 May 14;14(10):2065. doi: 10.3390/nu14102065 (PMC9146373; doi:10.3390/nu14102065)
Supplement: Supplementary file 1 [file nutrients-14-02065-s001.zip › nutrients-1687578-supplementary.pdf]

## Supplementary information

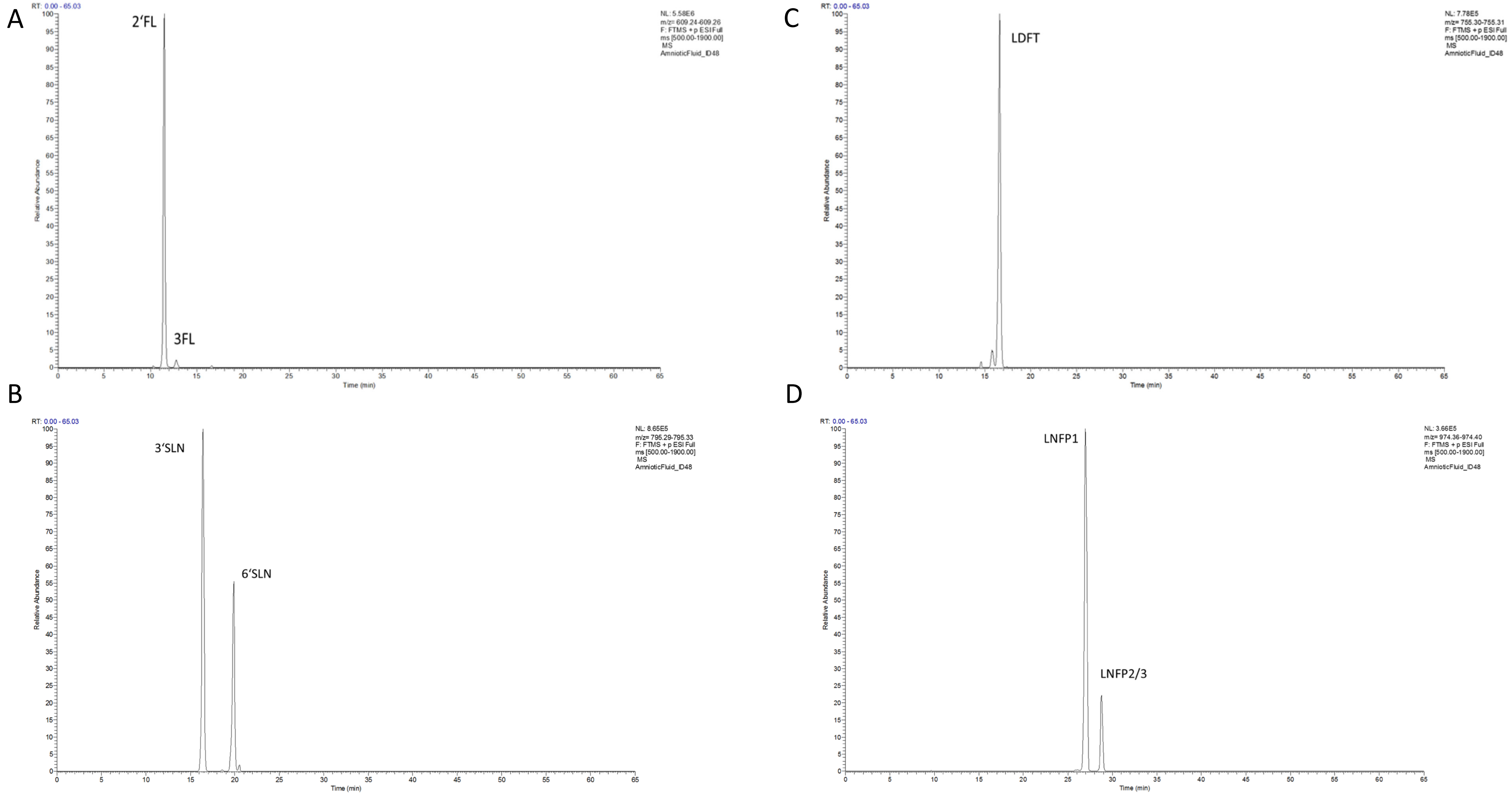
A
RT: 0.00 - 65.03
2'FL
3FL
NL: 5.58E6
m/z= 609.24-609.26
F: FTMS + p ESI Full
ms [500.00-1900.00]
MS
AmnioticFluid_ID48
Relative Abundance
Time (min)
C
RT: 0.00 - 65.03
LDFT
NL: 7.78E5
m/z= 755.30-755.31
F: FTMS + p ESI Full
ms [500.00-1900.00]
MS
AmnioticFluid_ID48
Relative Abundance
Time (min)
B
RT: 0.00 - 65.03
3'SLN
6'SLN
NL: 8.65E5
m/z= 795.29-795.33
F: FTMS + p ESI Full
ms [500.00-1900.00]
MS
AmnioticFluid_ID48
Relative Abundance
Time (min)
D
RT: 0.00 - 65.03
LNFP1
LNFP2/3
NL: 3.66E5
m/z= 974.36-974.40
F: FTMS + p ESI Full
ms [500.00-1900.00]
MS
AmnioticFluid_ID48
Relative Abundance
Time (min)

**Supplementary Figure S1.** Confirmation of human milk oligosaccharide (HMOs) identity by mass spectrometry.
Representative mass chromatograms of oligosaccharides isolated from amniotic fluid sample (36 wk of gestation) acquired by LC-MS/MS in multiple reaction monitoring mode are shown. Panels show the mass transitions 609/301 for 2-fucosyllactose (2'FL) and 3- fucosyllactose (3FL) (A), 795/325 for 3'-siallyllactosamine (3'-SLN) and 6'-siallyllactosamine (6'-SLN) (B), 755/301 for Lactodifucotetraose (LDFT) (C), 974/301 for lacto-N-fucopentaose 1 (LNFP1) and LNFP2/3 (D).

A

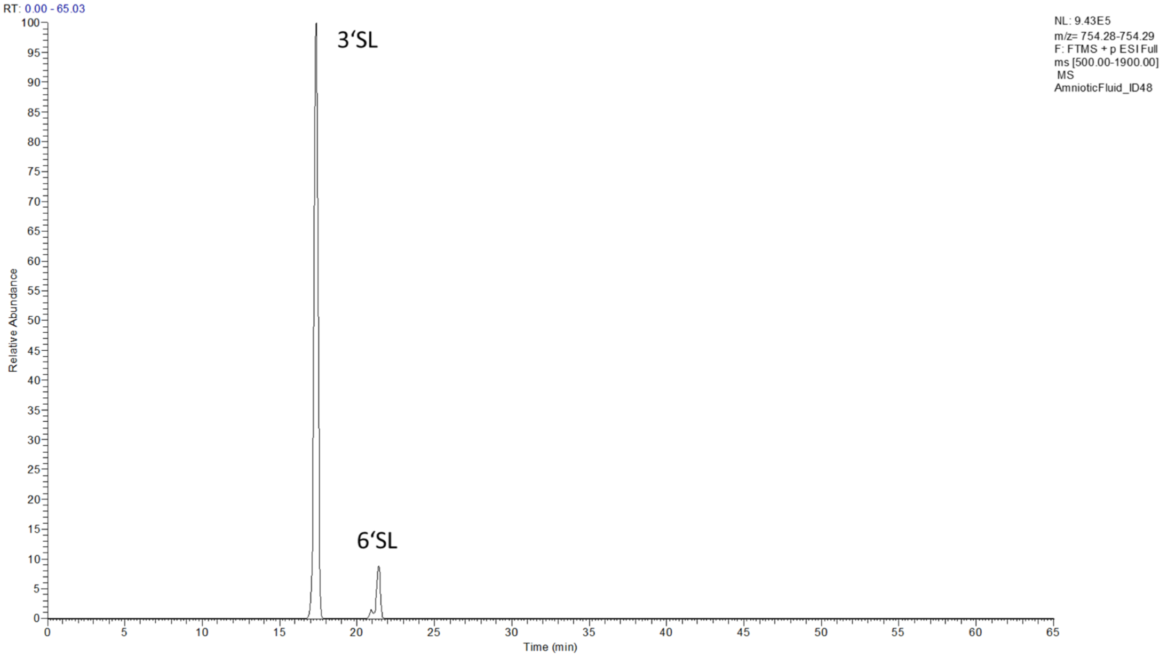


B

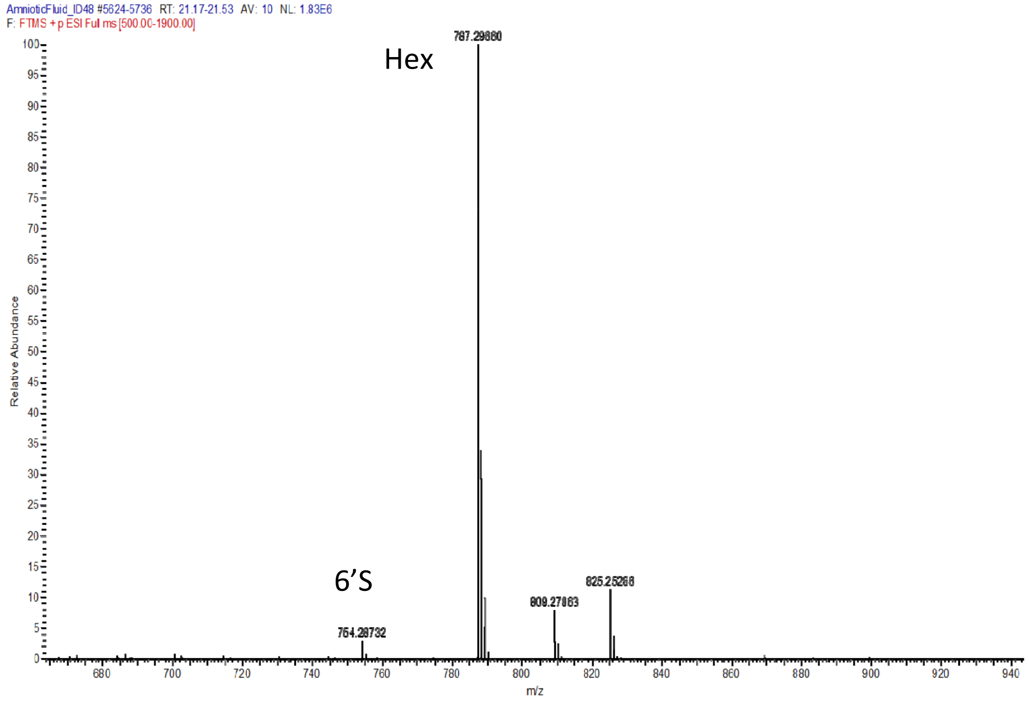

**Supplementary Figure S2**. Detection of an additional oligosaccharide co-eluding with 6’SL.
(A) Mass chromatogram of sialyllactose isolated from amniotic fluid sample (36 wk of gestation) acquired by LC-MS/MS in multiple reaction monitoring mode is showing the mass transition 754/301 for 3’-sialyllactose (3’-SL) and 6’SL. (B) Mass spectrum of the single peak at retention time of 6’SL shows a relatively higher signal with the mass of 787.29 corresponding to the 2AB H+ adduct of a tetrasaccharide containing 4 hexoses (Hex4).
